# Circular metagenome-assembled genome of *Candidatus* Cloacimonadota recovered from anaerobic digestion sludge

**DOI:** 10.1128/mra.00403-24

**Published:** 2024-06-25

**Authors:** Riku Sakurai, Yasuhiro Fukuda, Chika Tada

**Affiliations:** 1 Laboratory of Sustainable Animal Environment, Graduate School of Agricultural Science, Tohoku University, Osaki, Miyagi, Japan; 2 Japan Society for the Promotion of Science, Chiyoda-ku, Tokyo, Japan; Montana State University, Bozeman, Montana, USA

**Keywords:** anaerobic digestion, Cloacimonadota, metagenome

## Abstract

This study reports a circular metagenome-assembled genome (cMAG) of *Candidatus* Cloacimonadota recovered from a mesophilic full-scale food waste treatment plant. The cMAG spans 2,298,113 bp, with 980× coverage and 1 contig.

## ANNOUNCEMENT

*Candidatus* Cloacimonadota is a mysterious group of bacteria often detected abundantly in engineered systems, such as wastewater treatment plants and sludge bioreactors (1). Although the diversity and dominance of *Ca*. Cloacimonadota strongly imply their significance, the ecophysiological traits remain largely undetermined (2).

The first complete genome for this phylum was sequenced in 2008 (3). According to our current understanding, this study presents the second complete, circular metagenome-assembled genome (cMAG) of *Ca*. Cloacimonadota, which was recovered from a full-scale mesophilic food waste treatment plant in Tokyo, Japan in 2022 (4). The methodology of DNA extraction and metagenomic sequencing were mentioned in our previous study (4). DNA extraction was performed using a Fast DNA SPIN Kit for soil (MP Biomedicals, USA). For PacBio sequencing, we confirmed the quality of the DNA extract using the 5200 Fragment Analyzer System and the Agilent HS Genomic DNA 50 kb Kit (Agilent Technologies, USA). The library was constructed with the SMRTbell Express Template Prep Kit 2.0 (Pacific Biosciences of California). Sequencing polymerase was then bound to the SMRTbell libraries using the Binding Kit 2.2 (Pacific Biosciences of California). Adaptor sequences from the PacBio raw sequencing data were trimmed using SMRT Link version 10.1.0.119528. The HiFi reads were generated by aligning the trimmed reads with an average quality score of 20 or higher using pancake with KSW2 (5–7). For DNA nanoball sequencing (DNBSEQ), we prepared the library using the MGIEasy FS DNA Library Prep Set (MGI Tech). The quality was confirmed using the Fragment Analyzer and the dsDNA 915 Reagent Kit (Agilent Technologies). Circular DNA was then constructed using the MGIEasy Circularization Kit. Subsequently, DNA nanoballs were constructed using the DNBSEQ-G400RS High-Throughput Sequencing Kit (MGI Tech). Finally, 2 × 200 bp paired-end sequencing was carried out using the DNBSEQ-G400.

PacBio HiFi reads and DNBSEQ reads were hybrid assembled using metaSPAdes v.3.10.1 (8). The assembly was then merged with HiFi reads-only scaffolds [assembled by hifiasm_meta v. 0.3 (9)] using quickmerge v. 0.3 (10). Coverage was calculated by mapping DNBSEQ reads to the assembly using Bowtie2 v. 2.4.1 (11). Binning was performed by SemiBin2 v. 1.5.1 with the built-in model “wastewater” (12). Genome annotation was conducted using the DDBJ Fast Annotation and Submission Tool. Default parameters were used for all software unless otherwise noted.

**Fig 1:**
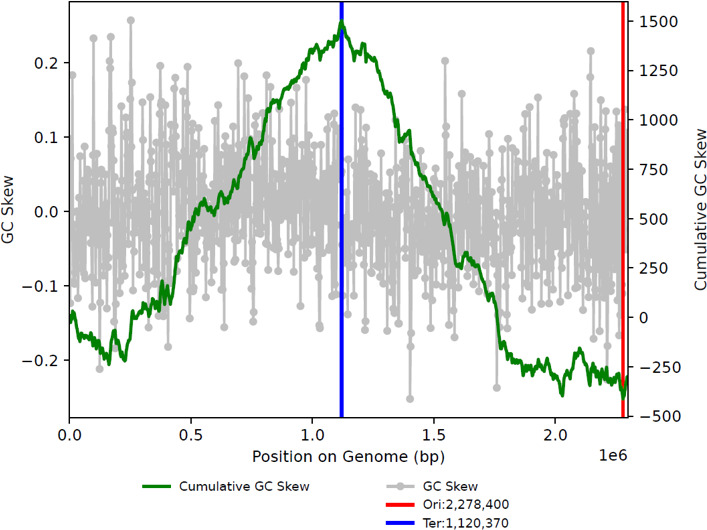
The GC skew analysis. The diagram indicates the GC skew (gray), cumulative GC skew (green line), Ori site (red line), and Ter site (blue line).

The resulting bin.919 consists of only one contig, with a completeness of 100% and contamination of 0% as assessed by MDMcleaner pipeline v. 0.8.7 (13). Circularization was achieved by connecting the 17,720-bp repeat regions detected at the beginning and end of the contig. GC skew was evaluated using the publicly available program gc_skew.py (https://github.com/christophertbrown/iRep) (14), revealing a well-defined pattern (15) (Fig. 1).

The recovered cMAG has a size of 2,298,113 bp, GC content of 52.4%, and 980× coverage. Taxonomic assignment by GTDB-Tk v2.3.2 (16) revealed that this cMAG was classified as d__Bacteria;p__Cloacimonadota;c__Cloacimonadia;o__Cloacimonadales;f__Cloacimonadaceae;g__UBA4175;s__UBA4175 sp002379855. There were 1,748 protein-coding sequences annotated, including two copies each of the 16S rRNA, 23S rRNA, 5S rRNA, and 6 tRNAs respectively. The cMAG also harbored six CRISPR arrays.

## Data Availability

Raw sequences and genome assembly are deposited under DDBJ BioProject accession number PRJDB17171. Raw reads and genome assembly are available under BioSample accession numbers SAMD00662589 and SAMD00747794, respectively.
